# Clinical significance of HIV-1 coreceptor usage

**DOI:** 10.1186/1479-5876-9-S1-S5

**Published:** 2011-01-27

**Authors:** Hanneke Schuitemaker, Angélique B van 't Wout, Paolo Lusso

**Affiliations:** 1 Department of Experimental Immunology, Sanquin Research, Landsteiner Laboratory, and Center for Infection and Immunity Amsterdam (CINIMA) at the Academic Medical Center of the University of Amsterdam, Meibergdreef 15, 1105 AZ Amsterdam, The Netherlands; 2 Laboratory of Immunoregulation, National Institute of Allergy and Infectious Diseases, National Institutes of Health, Bethesda, Maryland 20892, USA; 3 Present address: Crucell Holland BV, Leiden, The Netherlands

## Abstract

The identification of phenotypically distinct HIV-1 variants with different prevalence during the progression of the disease has been one of the earliest discoveries in HIV-1 biology, but its relevance to AIDS pathogenesis remains only partially understood. The physiological basis for the phenotypic variability of HIV-1 was elucidated with the discovery of distinct coreceptors employed by the virus to infect susceptible cells. The role of the viral phenotype in the variable clinical course and treatment outcome of HIV-1 infection has been extensively investigated over the past two decades. In this review, we summarize the major findings on the clinical significance of the HIV-1 coreceptor usage.

## Introduction

The entry of human immunodeficiency virus (HIV) into cells is critically dependent on the sequential interaction of the viral envelope with two cell-surface receptors, the CD4 glycoprotein and a 7-transmembrane-domain chemokine receptor such as CCR5 or CXCR4. The evolutionary choice of HIV of exploiting chemokine receptors as entry gateways has established a tight biological bond between HIV and the chemokine system, turning the natural ligands of these receptors into specific viral inhibitors. The first encounter between the fields of HIV and chemokines occurred unexpectedly at the end of 1995 with the discovery that three chemokines of the CC family, RANTES (CC-chemokine ligand 5 or CCL5), MIP-1α (CCL3) and MIP-1β (CCL4), act as potent and specific natural inhibitors of HIV-1 infection [1]. A few months later, in the spring of 1996, a totally independent experimental approach led to the identification of a chemokine receptor, CXCR4, as a critical cell-surface coreceptor for HIV-1 entry [2]. These two complementary findings triggered an authentic chain reaction of further breakthroughs, most notably the discovery of the second major HIV-1 coreceptor (i.e., CCR5), the identification of a specific chemokine ligand for CXCR4 (i.e., SDF-1/CXCL12), and the first definitive association of a genetic determinant (i.e., CCR5-Δ32) with HIV-1 resistance (reviewed in [3]). Looking backward, the exploration of this uncharted area of investigation has greatly advanced our understanding of the biology and pathogenesis of HIV infection, opening new perspectives for the development of effective measures for the therapy and prevention of AIDS.

## CCR5 and CXCR4: the two clinically relevant HIV-1 coreceptors

**Figure 1:**
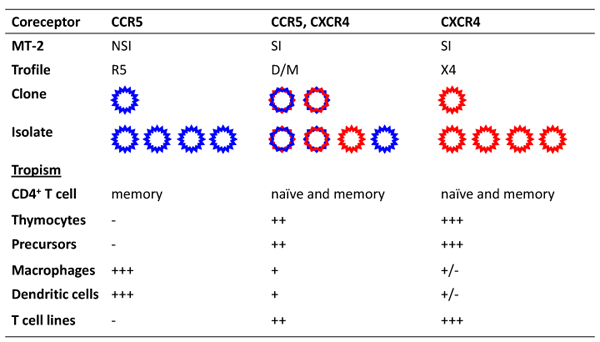
**Overview of coreceptor use and cell tropism of different HIV-1 variants.** Individual viral isolates are classified based on their ability to use CCR5 (R5 variants), CXCR4 (X4 variants) or both coreceptors (R5X4 variants). Bulk viral isolates capable of using both coreceptors are designated dual/mixed (D/M) as their quasispecies may contain any mixture of the various phenotypic variants. The cell tropism of each viral isolate is determined by the expression levels of CCR5 and CXCR4 on the various target cells.

Although several chemokine receptors may function as HIV-1 coreceptors* in vitro,* multiple lines of clinical and experimental evidence indicate that only two of them, CCR5 and CXCR4, have *bona fide* clinical relevance (reviewed in [3]). Both CCR5 and CXCR4 are expressed, in combination with CD4, on all the relevant target cells for HIV-1, including primary CD4^+^ T cells, macrophages and dendritic cells. Individual viral isolates are presently classified based on their ability to use CCR5 (R5 variants), CXCR4 (X4 variants) or both coreceptors (R5X4 variants) [4]). The dual-tropic R5X4 viruses are further classified as Dual-R (R5X4 variants with more efficient use of CCR5 than of CXCR4) or Dual-X (R5X4 with more efficient use of CXCR4 than of CCR5) [5-7]. In the absence of a more accurate characterization, bulk viral isolates capable of using both coreceptors are designated dual/mixed (D/M) as their quasispecies may contain any mixture of the various phenotypic variants (**Figure **1).

## *Ex vivo* determination of HIV-1 coreceptor usage

Before the identification of the coreceptors, HIV-1 isolates were characterized based on their ability to infect and induce syncytia (multinucleated giant cells) in CD4^+^ T-cell lines that express CXCR4 but not CCR5. Using the MT-2 cell line as a prototype, viruses that did not infect MT-2 cells were designated non-syncytium inducing (NSI), while viruses that did infect MT-2 cells were designated syncytium inducing (SI). Today, we attribute this differential ability to the viral coreceptor usage, and MT-2-positive variants are defined as either X4 or R5X4 [4]. Of note, the MT-2 assay can only detect CXCR4-using variants, and the absence of viral growth in MT-2 can be due either to the exclusive presence of R5 variants or to the failure to isolate HIV. For a precise determination of the coreceptor use of HIV-1 isolates, cell lines such as U87 and GHOST transfected with CCR5, CXCR4, or other coreceptors, have been used [8,9]. More recently, several recombinant phenotypic assays to determine coreceptor usage have been developed, such as the Trofile assay (Monogram Biosciences) [10]. Patient plasma is used to generate pseudoviruses or infectious recombinant viruses that include full-length or partial viral envelopes derived from the patient's virus population. The recombinants are subsequently tested on indicator cell lines expressing CD4 and either CCR5 or CXCR4. While the sensitivity of the Trofile assay for detecting CXCR4-using HIV-1 is similar to that of the MT-2 test [11], recombinant phenotypic assays are able to distinguish pure R5, D/M and pure X4 populations.

## HIV-1 coreceptor usage and genetic subtypes

The use of different coreceptors by HIV-1 is a general phenomenon that has been observed for viruses from all different genetic subtypes and circulating recombinant forms (CRFs) [12,13]. However, quantitative differences in the prevalence of CXCR4 usage among subtypes exist. Most of the initial studies on HIV-1 coreceptor usage evolution were performed on strains belonging to genetic subtype-B [14,15], which remains the best characterized subtype in terms of coreceptor usage. However, a more complex picture has been delineated with the extension of these studies to non-B HIV-1 subtypes.

One of the major discrepancies was the observation of a low frequency of CXCR4 usage among subtype-C strains [16-21]. Infection with subtype-C HIV-1 accounts for over half of the worldwide HIV-1 epidemics [22], and is as deadly as subtype-B infection. Although it has been suggested that the underrepresentation of CXCR4-using strains in these reports might be due to a sampling bias, later studies in treatment-experienced patients from Zimbabwe [23] and a recent study from South Africa have reported a higher prevalence of CXCR4-using variants than in the past [24], suggesting an ongoing evolution of the subtype-C HIV-1 epidemic in Africa (see also below).

An inverse skewing in coreceptor usage, with an increased presence of CXCR4-using strains, has instead been reported for subtype-D HIV-1 [5,12,25]. This observation is consistent with the faster pace of disease progression reported for subtype-D infection both in Africa [25-27] and outside Africa [28]. An increased rate of CXCR4 usage has also been reported in a study on a limited number of subtype-AE isolates [29,30]. Even more complex is the situation with mixed genotypes with only a few isolates so far characterized [31,32]. In a recent study in Guinea Bissau, an increasing and generally high frequency of CXCR4 tropism (86%) was observed in CRF02_AG [33].

## Molecular determinants of HIV-1 coreceptor usage

Studies conducted with subtype-B variants have shown that the HIV-1 coreceptor usage is determined by the viral envelope, primarily the second and third variable loops (V2 and V3) of the external HIV-1 envelope glycoprotein, gp120 [34-37]. Specifically, the presence of a positively-charged amino acid at either one or both of two specific positions in the V3 loop (positions 11 and 25) is strongly associated with a CXCR4-using phenotype in subtype-B primary isolates [38,39], suggesting that these amino acids play a crucial role in the interaction of gp120 with the coreceptors. Indeed, it has been shown that the V1V2 and V3 regions are involved in the interaction with CCR5 and CXCR4 [40-42]. This model is compatible with insights from structural studies in which V3 and a conserved coreceptor-binding site that includes the stem of the V1V2 loop are involved in coreceptor binding. In various trimeric models of the gp41-gp120 envelope glycoprotein complex, V2 directly interacts with V3 and both participate in coreceptor binding [43-46].

As shown for subtype-B HIV-1, the V3 loop also seems to be the principal genetic determinant of the coreceptor choice among subtype-D (10 variants studied) and subtype-A (one variant studied) [12]. In later studies, subtype-C CXCR4-using strains did not show the same dependence as other subtypes on positively-charged residues in the V3 loop [47,48].

## Predictive algorithms of HIV-1 coreceptor usage

The identification of viral genotypic changes associated with different coreceptor usage has led to the development of sequence-based algorithms to predict coreceptor usage. Starting with the 11/25 rule derived from subtype-B variants [39], efforts have concentrated mainly on identifying sequence patterns in the V3 loop. Most genotypic predictors of coreceptor usage incorporate information from across the V3 region (**Table **1) [49-53], in some cases along with genotypic correlates outside the V3 [54]. Some methods also incorporate clinical data [55]. Progress is also being made on the inclusion of structural information to assist prediction [56], as well as on the ability to discriminate between X4 and R5X4 virus [57]. On cloned viruses belonging to genetic subtype B, the specificity and sensitivity of most predictive methods exceed 90% and 80%, respectively. However, the sensitivity drops below 50% when bulk uncloned sequences and non- subtype B viruses are assayed. Moreover, technical limitations to the generation of unambiguous DNA sequences from the HIV-1 envelope region that has insertions and deletions that prevent the generation of interpretable electropherograms, interfere with a predictive determination of tropism in a significant fraction of patient samples. This in combination with the limited predictive power obviously has implications for a clinical diagnostic application of bulk sequencing technology and current predictive algorithms for HIV-1 coreceptor usage. To date, studies of genotypic predictors have been retrospective with patient samples selected based on availability of phenotypic tropism determinations. Prospective studies will be needed to firmly establish the clinical usefulness of genotypic tropism determination.

**Table 1:** Bioinformatic predictors of HIV-1 coreceptor use based on V3 loop sequence.

Predictor	URL	Method	Ref
R5/X4 and NSI/SI network	http://cancer.med.unc.edu/swanstromlab/resources.html	Neural network	Resch et al, 2001
WebPSSM	http://indra.mullins.microbiol.washington.edu/webpssm/ http://fortinbras.us/cgi-bin/fssm/fssm.pl	PSSM	Jensen et al, 2003
WetCat	http://genomiac2.ucsd.edu:8080/wetcat/v3.html	SVM	Pillai et al, 2003
Geno2pheno	http://coreceptor.bioinf.mpi-inf.mpg.de/	SVM	Sing et al, 2007
V3SD	The source code for prediction and analysis is available upon request.	SVM	Sander et al, 2007
R5/X4-pred	http://yjxy.ujs.edu.cn/R5-X4 pred.rar	Random forests	Xu et al, 2007
ANN	Not available	Neural network	Lamers et al, 2008
hiv-dskernel	http://genome.ulaval.ca/hiv-dskernel	SVM	Boisvert et al, 2008

As discussed below, the selective pressure that leads to the emergence of CXCR4-using strains is complex and variable, and clinically relevant CXCR4-using minorities may coexist with a predominant R5 virus, even though they remain rare enough to go undetected by sequencing [58]. In addition, not all determinants of coreceptor usage lie within the V3 loop, the region employed by most current predictors [35,59]. Accurate prediction is also complicated by the fact that the V3-C4 region of the envelope gene, which has the greatest influence on tropism, also has a relatively high rate of diversity although the V3 region itself does contain conserved segments [60]. This complicates the interpretation of sequences, especially when so-called bulk sequencing is employed. This method captures only the most prevalent sequence (>25% of the total quasispecies), and the results become difficult to interpret when many genetic variants are present simultaneously. However, continuous progress is being made, especially with the application of next generation sequencing [61].

Clinical trials with CCR5 antagonists have indicated that patients with detectable CXCR4-using virus are unlikely to show a significant decrease in viral load in response to CCR5 antagonists [62,63]. Therefore, prior to initiating treatment with CCR5 inhibitors, patients are now screened to exclude those who harbor CXCR4-using variants. Both phenotypic and genotypic assays have been developed for this screening, but the Monogram Trofile assay is currently the only clinically validated test and has been used to screen the largest number of patients [10,64]. As more combined V3 genotype-phenotype data become available, genotypic predictors of coreceptor usage are likely to become a viable alternative to phenotypic assays. Indeed, recent data from the HOMER cohort and the MOTIVATE and MERIT trials show that genotype-based methods performed on populations-based samples, or “bulk” sequence data, are equivalent to the Trofile assay and that deep sequencing can actually improve the phenotypic results [65,66].

## Coreceptor usage in primary HIV-1 infection

### Coreceptor usage and HIV-1 transmission

Despite an extensive literature on the subject, the process of* in vivo* transmission of HIV-1 remains largely unknown and most of the current models are essentially conjectural. In fact, there are virtually insurmountable difficulties in studying the earliest events of HIV-1 infection in the human species, while nonhuman primate models, albeit useful, provide only a partial and, in all likelihood, non-physiological picture. In fact, most of the observations on the acute phase of HIV-1 infection in humans are made several weeks or even months after the initial transmission event, typically in subjects who manifest clinical signs of acute retroviral syndrome. With this caveat in mind, the bulk of evidence indicates that R5 HIV-1 variants are largely prevalent during the acute phase of infection [67,68]. Even if both R5 and CXCR4-using variants are present in the donor, most often only the R5 variants are detected in the recipient [69-71]. Whether this early R5 predominance reflects a* bona fide* transmission bias or a superior* in vivo* fitness of R5 strains during the early phase of infection remains uncertain.

Several studies have attempted to correlate the predominance of R5 HIV-1 strains during the acute phase with a biological bottleneck inherent to the genital mucosa, variously related to trapping and inactivation of CXCR4-using virus by mucin and innate antiviral proteins, preferential transcytosis of R5 viruses in endothelial cells and/or preferential amplification of R5 viruses by resident macrophages, dendritic cells and/or Langerhans cells (reviewed in [72]. In reality, however, no conclusive evidence has been provided to indicate that CXCR4-using strains are less able or unable to sustain mucosal transmission. For example simian/human chimeric immunodeficiency viruses (SHIV) bearing an X4 HIV-1 envelope can be readily transmitted via the mucosal route in macaques, and have widely been used as a reference model [73].

Another important element that is rarely taken into consideration in the HIV-1 transmission equation is the transmitter bias, due to the fact that people with replicating CXCR4- using viruses may be in a more advanced stage of their disease and less prone to engage in risky sexual behavior [74]. If a majority of transmissions occur from asymptomatic individuals who at that time still only harbor R5 variants, then this would indeed contribute to the scarcity of transmission of CXCR4-using variants.

### Post-transmission events

Another potential contributing factor in the marked R5-variant predominance during acute primary HIV-1 infection is a post-transmission amplification bias. In peripheral blood, the high proportion of CCR5^+^CD4^+^ T cells that are recruited during acute HIV- 1 infection may favor R5 variants to replicate and thereby outcompete putative co-transmitted CXCR4-using variants. Moreover, the high proportion of memory/activated CCR5^+^CD4^+^ T cells present in the mucosal-associated lymphoid tissue, which is considered a major site of HIV-1 replication during primary infection [75], may provide the optimal environment for preferential R5 HIV-1 amplification. Of importance, these cells also express high levels of integrin α4β7, an important facilitator of HIV-1 infection [76].

Although primary infection with CXCR4-using HIV-1 strains is believed to be a rare event, mixed R5/X4 primary infections have been clearly documented in a few patients studied longitudinally from an early stage post-infection [77,78]; interestingly, however, the CXCR4- using component was selectively cleared from plasma with the transition to the chronic phase in which only R5 variants could be recovered, raising the possibility that mixed R5/X4 transmission may in fact be more frequent than it appears, albeit underestimated due to late sampling, subsequent to the disappearance of the CXCR4-using component from plasma. In a recent retrospective evaluation of a large number of patients (n = 390) enrolled in the PRIMO cohort in France between 1996 and 2007, a relatively high prevalence (15.9%) of predicted CXCR4-using viruses was documented during primary infection [79]. However, subsequent phenotypic analysis of the patients in this study infected with non-subtype B variants (n=131) showed that genotypic predictions overestimated the proportion of CXCR4-using variants in non-subtype B infected patients, resulting in a much lower prevalence (0.8%) of actual CXCR4-using variants [80]. In agreement, a low prevalence of CXCR4-using viruses (4-6%) was observed in three recent cohorts of seroconverters: one from the United States (n=150) during the period 1999-2003 [81], one from France (n=133) during the period 1995-2008 [82], and one from the Netherlands (n=46) during the period 2003-2008 (ABW et al., unpublished observations). Moreover, detailed investigations of individuals experiencing primary infection and sampled prior to seroconversion (Fiebig stage I-V) using single genome amplification have revealed a consistent pattern of CCR5 dependence at this stage [67,68,83-88] indicating that any putative post-transmission amplification bias would have to occur within the first few days of exposure.

### Protective effect of congenital CCR5 deficiencies

The most convincing argument in favor of the *bona fide* predominance of R5 variants in the initial transmission events is the high degree of protection from HIV-1 infection conferred by genetic deficiency of CCR5. Subjects that are homozygotes for the* CCR5-Δ32* allele are highly protected from HIV-1 infection [89]. Indeed, this genotype is enriched among individuals who remain seronegative despite high-risk sexual behavior [90,91] as well among hemophiliacs who had remained HIV negative despite exposure to batches of clotting factor that were known to be the cause of HIV infection in other hemophiliacs [92]. CCR5wt/Δ32 heterozygosity does not confer protection from HIV-1 infection, but this genotype has been associated with a significantly lower viral set point and delayed disease progression [93-96]. A second crippling polymorphism, m303, that introduces a premature stop codon in the CCR5 gene has been identified in exposed-uninfected subjects [97].

Despite the strong protective effect conferred by congenital CCR5 deficiencies, a handful of infected* CCR5-Δ32* homozygotes have been reported, all invariably harboring CXCR4- dependent HIV-1 strains [98]. Interestingly, two* CCR5-Δ32^+/+^* individuals were found to harbor an inherently (not mixed) dual-tropic virus (R5X4) that remained stable over time [99], suggesting that maintenance of the CCR5-using envelope conformation might provide a selective advantage* in vivo* despite the absence of a usable receptor. Although the viral load in infected *CCR5-Δ32^+/+^* subjects tends to be low, a rapid depletion of circulating CD4^+^ T cells has been noted [98]. However, the very limited number of individuals characterized thus far makes it difficult to evaluate whether the clinical and virological course of an X4 HIV-1 infection *d'emblée* differs from that of conventional R5 HIV-1 infection in individuals with wild-type *CCR5* genes. In this respect, viral variants with X4/DM phenotype have been detected in individual case reports of acute HIV-1 infections with peculiarly severe clinical manifestations [100-103], but again their number is too limited to draw any reliable conclusions and it is difficult to exclude other confounding cofactors.

### Role of minor coreceptors

Usage of the so-called “minor” coreceptors during primary HIV-1 infection has only marginally been investigated. Interestingly, a high frequency of CCR3 usage during primary infection has been detected by direct cloning and expression of viral sequences, while* in vitro* culture apparently selects against CCR3 usage leading to the expansion of variants with exclusive CCR5 usage [104]. By contrast, in a few cases of primary infection with subtype- C HIV-1 studied, all of R5 phenotype, there was a surprisingly high frequency of usage of GPR15, APJ and CXCR6, but not of CCR3 [105,106]. Further studies are warranted to address the relevance of minor coreceptors in HIV-1 transmission.

## Coreceptor usage in chronic HIV-1 infection

### In vivo evolution of HIV-1 coreceptor usage

**Figure 2:**
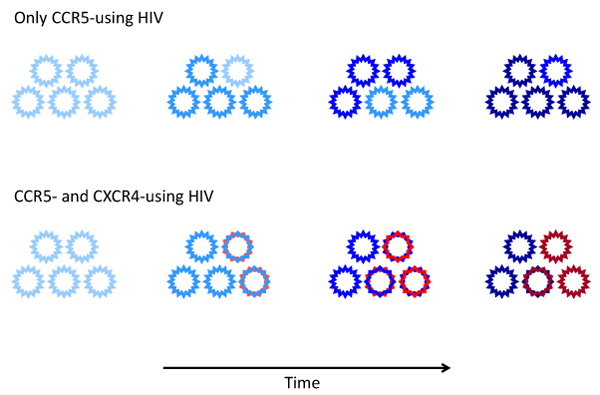
**Evolution of HIV-1 coreceptor usage during the progression of the disease.** In most individuals HIV-1 infection is initially sustained by CCR5-using variants (R5, blue). In ~50% of the patients infected with subtype-B HIV-1, the CCR5-using variants acquire the ability to use CXCR4 (R5X4, blue/red) prior to AIDS diagnosis. Subsequently, these dual-tropic R5X4 variants may lose their ability to use CCR5 (X4 variants, red). Over time, viral coreceptor usage evolves resulting in viral variants with increased affinity for their respective coreceptor (increased color intensity).

During the asymptomatic phase of HIV-1 infection a homogeneous R5 virus population is commonly present that generally has the ability to replicate efficiently in both T cells and macrophages [70,71]. In many patients, a typical pattern of viral evolution has been documented during the course of the infection with the emergence, usually in concomitance with the earliest signs of disease progression, of CXCR4-using variants. However, this pattern is not consistently observed in all patients progressing to AIDS (**Figure **2). For example, in patients infected with subtype-B HIV-1, variants that use CXCR4 can be isolated from approximately half of the patients who have developed AIDS [15,24,33,106-109]. Of note, such variants may first appear during the asymptomatic phase of infection, before AIDS is diagnosed [106], albeit after an initial decline of CD4^+^ T cells [110]. Patients progressing to AIDS often harbor viral populations that can use multiple coreceptors including CCR5, CXCR4 and one or more minor coreceptors [15]. Whether a promiscuous coreceptor usage provides a selective advantage for HIV remains uncertain since most of the minor coreceptors show a low and/or tissue-specific expression pattern.

Until recently, the general consensus was that CXCR4-using variants only emerge in a certain proportion of HIV-1 infected individuals, which varies according to the viral genetic subtype. However, recent evidence has challenged this view suggesting that the prevalence of CXCR4 usage is continuously evolving over time. Data from the Amsterdam Cohort Studies on HIV infection and AIDS (ACS) show a continuously ongoing X4 conversion rate at the population level, even after AIDS diagnosis (H.S. et al., unpublished results). Thus, it seems that the emergence of CXCR4 usage is a matter of time and that some HIV-1 infected individuals die before they develop CXCR4-using variants. Molecular cloning of the viral quasispecies has documented the frequent presence of DNA sequences predictive of CXCR4 usage in blood cells even in the absence of detectable replication of such variants, as well as transient appearances of CXCR4-using strains on a background of sustained R5 persistence [50,111,112]. These observations further challenge the simplistic concept of a single and irreversible coreceptor- switching event, depicting a complex dynamic state where variant predominance is a continuously evolving process governed by multiple interactive factors. After the appearance of CXCR4-using variants, R5 variants remain present in the vast majority of patients [108,113]. Pure X4 virus populations are rather infrequently detected and often restricted to late-stage disease [114], although they have been documented earlier in rare cases of* ab initio* X4 transmission [70,115,116] often associated with the absence of CCR5 expression in the host [98].

### Origin of CXCR4-using HIV-1 variants

Several lines of evidence suggest that CXCR4-using variants evolve* in vivo* from pre-existing R5 variants. Phylogenetic analysis of longitudinally obtained HIV-1 variants from individual patients has shown that although R5 and X4 variants from a single patient form separate monophyletic clusters in an unrooted tree, they do cluster together when an appropriate outgroup is used. Although this implies that R5 and CXCR4-using variants from a single individual are more related to each other than to HIV-1 variants from another person, it does not exclude that both variants may be initially transmitted together and then differentially expressed* in vivo,* with CXCR4-using variants maintained in a cryptic condition until immune deterioration occurs. However, the first detectable CXCR4-using variants are more closely related to the R5 variants obtained at the same time than co-existing R5 and CXCR4-using variants derived at later time points. Moreover, recently emerged CXCR4-using variants generally maintain the ability to use CCR5 and in phylogenetic trees these dual-tropic R5X4 populations cluster between pure R5 and later-stage CXCR4-using virus populations derived from the same individual. These observations are compatible with the hypothesis that the first CXCR4-using variants evolve from pre-existing R5 variants within the same individual, rather than remaining silent for many years after an initial independent transmission.

### Clinical significance of CXCR4-using HIV-1 variants: cause or consequence of immune deterioration?

**Figure 3:**
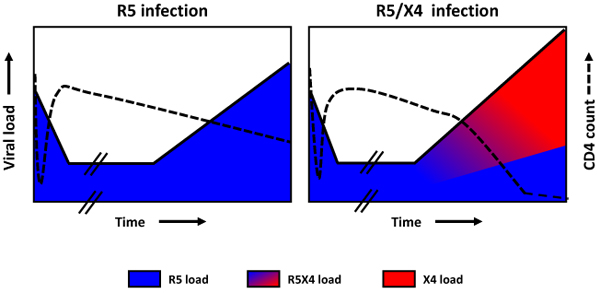
**Typical course of HIV-1 disease in relation to HIV-1 coreceptor usage.** (**A**) Individual infected with pure R5 variants: constant rate of CD4 decline and viral load incline. (**B**) Individual with emergence of CXCR4-using variants in the course of infection: accelerated CD4 decline, viral load incline and disease progression upon emergence of CXCR4-using variants. While CXCR4-using variants can emerge at any stage of infection, untreated individuals who develop such variants progress to AIDS within an average of 2 years after their first detection.

**Figure 4:**
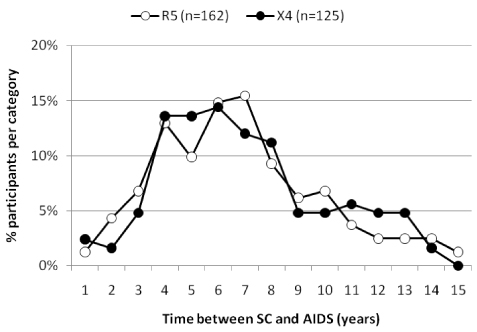
**Time between seroconversion (SC) and AIDS among participants in the Amsterdam Cohort Studies on HIV infection and AIDS with or without CXCR4-using variants detected prior to AIDS diagnosis.** All homosexual and drug-user participants with AIDS diagnosis according to the 1993 CDC definition prior to initiation of effective antiretroviral therapy were included in the analysis. Participants were divided into 2 groups based on the presence (X4, closed circles) or absence (R5, open circles) of CXCR4-using HIV-1 variants as detected by the MT-2 assay prior to AIDS diagnosis. Time between SC and AIDS was categorized in 12-monthly intervals. The graph shows the proportion of participants per category as a percentage of the total number of participants in the group.

The emergence of CXCR4-using HIV-1 variants in a patient is almost invariably associated with a subsequent increase in the rate of decline of circulating CD4^+^ T cells, an accelerated disease progression, and a poor prognosis for survival [106] (**Figure **3). Indeed, studies in the era preceding the introduction of combination antiretroviral therapy (cART) demonstrated a significant acceleration in the rate of CD4^+^ T-cell decline after the first detection of CXCR4-using variants [106,107]. However, the presence of CXCR4-using variants is not an obligatory prerequisite for disease progression and a significant proportion of individuals progress to AIDS and AIDS-related death while harboring exclusively R5 HIV-1 variants. Notably, the time from seroconversion to AIDS is not significantly different between individuals who harbor only R5 variants as compared to individuals with detectable CXCR4-using variants (**Figure **4).

The accelerated CD4^+^ T-cell decline after the first appearance of CXCR4-using HIV-1 variants seems to be determined by the greater proportion of CD4^+^ T cells that express CXCR4, which provides a larger target-cell pool for the virus. Indeed, up to 90% of CD4^+^ T cells express CXCR4 whereas CCR5 expression is restricted to a smaller subset (15-35%) that express a memory/activated phenotype [117-119]. Specifically, CXCR4, but not CCR5, is expressed on naïve CD4^+^ T cells [118], a large population of non-antigen-primed T cells. Thus, naïve CD4^+^ T cells are exclusively a target for CXCR4-using variants [120]. The pathological consequences of infection of naïve CD4^+^ T cells may be dramatic, resulting in the depletion of the largest pool of CD4^+^ T cells in the body. Considering the potential beneficial effect of an expanded target-cell population, it is puzzling that CXCR4-using variants emerge in only a proportion of infected individuals [106] even though a limited number of amino acid substitutions in V3 is sufficient to confer CXCR4-using capability to R5 variants [38,121].

It has been hypothesized that the absence of CXCR4-using variants early in HIV-1 infection may be due to their higher vulnerability to the host adaptive immune responses, in particular neutralizing antibodies. The fact that these variants appear more frequently after the initial decline of CD4^+^ T cells indeed suggests that they may represent a peculiar, congenic form of opportunistic infection. In a recent study, we found that the first appearing CXCR4-using variants are more sensitive to neutralizing antibodies directed against the CD4 binding site than their co-existing R5 variants [122]. A greater sensitivity to both monoclonal antibodies and sera from infected patients was also documented in sequential isolates derived from a cohort of perinatally-infected children (P.L. et al., unpublished data). Further evidence for a greater sensitivity of CXCR4-using variants to antibody-mediated neutralization has come from the characterization of a conserved neutralization epitope within the V3 domain of gp120: such epitope, designated D19, is invariably cryptic in R5 variants of different genetic subtypes, but it is consistently exposed in CXCR4-using variants, rendering such variants sensitive to neutralization by a specific antibody [123]. A role of cell-mediated immune responses was instead suggested by the re-emergence of CXCR4-using strains in dually-infected (R5+X4 SHIV) macaques after* in vivo* depletion of CD8^+^ T cells [124]. Additional data pointing to a lower* in vivo* fitness of CXCR4-using variants have come from the results of clinical trials with the CCR5- antagonist maraviroc (MVC). Resistance to MVC was shown to develop by two mechanisms: a reduced drug susceptibility associated with changes in the V3 loop that allow the R5 virus to use CCR5 in its MVC-bound conformation [125] or the emergence of variants that use CXCR4 [58]. In the latter scenario, the CXCR4-using virus seems to originate from an unrecognized pretreatment reservoir, indicating that screening assay sensitivity remains to be improved. Interestingly, however, the circulating virus was shown to revert to the R5 phenotype following cessation of MVC, indicating that the selective pressure acting against CXCR4 usage was preserved.

Altogether, the above observations suggest that effective host immune responses may exert a selective pressure that hinders the emergence of CXCR4-using variants. However, when the host immune competence begins to fade during the progression of HIV-1 disease, such pressure would start to wane, paving the way for the emergence of CXCR4-using variants. Although the host immune surveillance may explain, at least in part, the absence of CXCR4- using variants during the asymptomatic phase of HIV-1 infection, it cannot justify the apparent scarcity of CXCR4-using variants in the very early phase of infection when neutralizing antibodies and cytotoxic T lymphocytes are still absent. As discussed above, this may result from a lack of transmission or to the inability of recently transmitted CXCR4-using virus to compete with co-existing R5 HIV-1 during the earliest phase of infection.

### R5 HIV-1 evolution in AIDS_progressors without coreceptor switch

As stated above, a significant fraction of patients progresses to full-blown AIDS without experiencing an overt switch to CXCR4 usage. However, accumulating evidence indicates that in spite of their ''monogamous'' CCR5 use late isolates from these patients are inherently more pathogenic [126] and RANTES-resistant than early isolates [127,128]. In line with these observation is the ability of late-stage CCR5-restricted HIV-1 variants to use chimeric coreceptors in which parts of CCR5 have been replaced with segments of CXCR4 (R5 broad), whilst early CCR5-using HIV-1 variants are restricted to the use of wild-type CCR5 (R5 narrow) [127,129,130].

This* in vivo* evolution of CCR5-restricted HIV-1 in humans is similar to that observed in nonhuman primates infected with SIV, which never acquires CXCR4 usage even though its pathogenicity increases during the late disease stages [131]. Moreover, we have observed that the ability of R5 isolates to replicate in macrophages is progressively reduced during the course of infection, resulting in a predominantly T-cell tropic R5 HIV-1 quasispecies even before the progression to AIDS [108]. In about half of subtype-B HIV-1 infected individuals, this shift towards full T-cell tropism precedes the emergence of CXCR4 using HIV-1 variants.

### R5X4 HIV-1 variants as an intermediate evolutionary stage

Evidence suggests that the evolutionary changes in the V3 loop involved in the coreceptor-usage switch are gradual and accretive, and that dual coreceptor usage (R5X4) represents an intermediate transitional phase. R5X4 viruses can be more efficient in using either CCR5 (dual-R) or CXCR4 (dual-X), or can use both coreceptors with similar efficiency [5-7]. As mentioned above, there is a continued evolution in viral coreceptor usage* in vivo,* resulting in a broad range of coreceptor affinities within the HIV-1 quasispecies. The limited number of amino acid substitutions required to confer CXCR4-using capability to R5 variants* in vitro *[38,121], combined with the high mutation rate of HIV-1 and the larger population of target cells expressing CXCR4, would predict an early and nearly universal emergence of CXCR4-using variants during chronic infection. However, the fact that CXCR4-using variants seem to develop successfully only once during HIV-1 infection [112,132], the scarcity of R5 virus variants with intermediate genotypes, and the fact that the newly emerged CXCR4-using variants differ from coexisting R5 variants by more than the minimally required number of amino acid mutations [133] altogether suggest that the virus evolves to the CXCR4-using phenotype through less-fit intermediate stages. The late emergence of CXCR4- using variants might be explained by an inability of these intermediate variants to compete with the well-established R5 virus population in spite of their broader cellular host range. Once established, however, R5X4 viruses may have developed the optimal fitness to predominate during the transition phase [134], although they may eventually be outcompeted by HIV-1 variants with a pure X4 phenotype.

### Evolution of co-existing R5 and CXCR4-using HIV-1 variants

The progressive divergence between co-existing R5 and CXCR4-using clones in phylogenetic trees reflects the continuous evolution of the variable loops of gp120 following the acquisition of CXCR4 usage. This implies that the structure of gp120 continues to evolve to optimize its interaction with the coreceptors. This evolution is accompanied by improved coreceptor-binding affinity, which in turn is reflected in a decreasing sensitivity of R5 variants to inhibition by CCR5-binding chemokines and small- molecule CCR5 antagonists [127,128,135,136], as well as of CXCR4-using variants to SDF-1 and the synthetic antagonist AMD3100 [137].

Once established, CXCR4-using variants are likely to be more replication competent than R5 variants, given the broader target-cell range and the generally higher replication kinetics of CXCR4-using HIV-1* in vitro *[132,138,139]. In light of these considerations, it seems paradoxical that R5 variants persist and may even expand* in vivo* after the emergence of CXCR4- using HIV-1 [113]. The most likely explanation for the observed coexistence of R5 and CXCR4- using variants is their distinct target cell range within an infected patient [120,140,141]. R5 variants seem to reside mainly in activated/memory CD45RO^+^CD4^+^ T cells, which express CCR5, whereas CXCR4-using variants reside in both CD45RA^+^ and CD45RO^+^CD4^+^ T cells [120]. Interestingly, evidence for frequent recombination events between co-existing R5 and CXCR4-using variants has been reported [142,143]. While phylogenetic trees based on envelope sequences show a clear-cut separation of coexisting R5 and CXCR4-using virus variants, this separation is not observed when the phylogenetic tree is based on* gag* sequences. Apparently, R5 and CXCR4-using variants can co-infect cells that express both coreceptors, thus allowing for the recombination of their genetic material.

Interestingly, the distribution of R5 and CXCR4-using variants in different blood compartments may vary. Two recent studies reported a higher prevalence of predicted CXCR4-using envelopes in PBMC than in plasma [144,145], although a third study could not confirm this discrepancy [146]. Differences in the sensitivity of the coreceptor usage predictors used may be responsible for the different study outcomes. Thus, the relevance of the choice of patient material for the determination of HIV-1 coreceptor usage remains to be established.

## Evolutionary dynamics of coreceptor usage in different HIV-1 subtype epidemics

**Figure 5:**
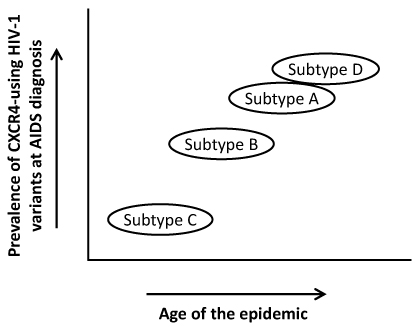
**Hypothetical model of the evolutionary dynamics of CXCR4 usage in relation to the age of each HIV-1 subtype epidemic.** Recent data suggest that the proportion of patients with detectable emergence of CXCR4-using HIV-1 variants varies in relation to the age of the different subtype epidemics, with subtype D (high CXCR4 switch rate) being the oldest and subtype C (low CXCR4 switch rate) the youngest.

The different prevalence of CXCR4-using variants among different HIV-1 genetic subtypes remains puzzling. As CXCR4-using variants emerge after an accumulation of mutations, the different prevalence observed among different subtypes and CRFs may reflect the same phenomenon at the population level [147], although a direct relationship between evolutionary rate and development of CXCR4 usage has not been specifically investigated. Based on a series of recent observations, however, it is tempting to speculate that the prevalence of CXCR4-using HIV-1 is increasing with the age of the subtype epidemics (**Figure **5). Indeed, phylogenetic studies have revealed that the subtype-D epidemic, which has the highest prevalence of CXCR4- using variants, is one of the oldest, while the subtype-C epidemic, which has a much lower prevalence of CXCR4-using variants, is considered one of the most recently emerged [148,149] (UNAIDS). The subtype-B HIV-1 epidemic has an intermediate pattern, both in terms of age and prevalence of CXCR4-using HIV-1 [150]. This assumption is highly speculative and not supported by all the data available at present. However, if confirmed, it would imply that all the subtype epidemics are evolving towards a higher prevalence of CXCR4-using HIV-1 variants although it is conceivable that each epidemic might reach a point of equilibrium beyond which such prevalence will not further increase.

## HIV-1 coreceptor usage and antiretroviral therapy

Early studies of zidovudine monotherapy showed that the beneficial effect was mainly limited to persons who do not develop CXCR4-using variants [151,152]. Moreover, a trend towards increased emergence of CXCR4-using variants under zidovudine therapy was observed [151]. Further studies confirmed a differential efficacy of certain HIV-1 reverse transcriptase inhibitors against R5 and CXCR4-using viruses, with zidovudine showing a higher efficacy against R5 viruses and didanosine against CXCR4-using viruses [153,154]. This could be explained by the differential efficiency of activation of zidovudine and didanosine in CCR5-expressing activated memory T cells and CXCR4-expressing naive and resting memory T cells. Drugs that are activated equally in both types of target cells, such as lamivudine, or that did not require activation, such as the protease inhibitor (PI) ritonavir, showed equal activity against both variants [154,155].

Although several studies have reported differential responses to single-agent ART according to coreceptor usage of the HIV-1 variant present in the patient, the association between clinical efficacy of currently used cART regimens and coreceptor usage has not been rigorously evaluated. In 80-90% of asymptomatic treatment-naïve patients, only R5 virus is found [156-159], whereas recent cross-sectional studies demonstrated CXCR4-using virus in 40-55% of patients with previous antiretroviral exposure [114,159-161] , probably reflecting the generally lower CD4 counts in individuals who are eligible for initiating cART [162].

Once patients are started on cART, both increased and decreased frequencies of CXCR4- using viruses have been reported [23,163-167], but neither seems to predict treatment success [168]. In patients experiencing virological failure under ART, HIV-1 tropism shifts in either direction (R5>X4 or X4>R5) have been reported in 13.2%–28.6% of subjects [162,169]. For suppressive cART, an early study reported rates of tropism shifts as high as 37.5% [167], but most recent evidence suggests that HIV-1 tropism shifts under suppressive ART are rare (3- 11%)[146,170].

While initial data from the HOMER cohort showed that baseline sequences predictive of CXCR4 usage were associated with increased risk of clinical progression during cART [171], the presence of CXCR4-using variants at baseline was not predictive of survival or treatment response after adjusting for other baseline parameters [172,173]. Instead, it was significantly associated with lower CD4 counts regardless of antiretroviral treatment exposure. Similar results were recently obtained both in a UK cohort [174] and in a Dutch cohort (ABW et al, unpublished results). It is still unclear whether the effectiveness of different regimens (e.g. PI containing or not) varies according to the coreceptor usage of the HIV-1 variants harbored by the patient at baseline.

## Conclusion

The identification of two major HIV-1 coreceptors, CCR5 and CXCR4, the finding of their differential expression on various HIV-1 target cells, and the discovery of viral variants with differential ability to use them have significantly advanced our understanding of the clinical course of HIV-1 infection and the efficacy of antiviral therapy. The establishment of a connection between HIV-1 and the chemokine system has resulted in the development of a new drug class that directly interferes with CCR5 usage by HIV-1. These drugs not only increase the range of therapeutic options for the systemic treatment of HIV-1-infected individuals, but can also be employed as topical microbicides to prevent HIV-1 acquisition at the mucosal level. Despite these extraordinary successes, many questions regarding the clinical significance of HIV-1 coreceptor usage remain unanswered. Finding an answer to these questions may pave the way toward a deeper understanding of AIDS pathogenesis and a more effective control of the HIV-1 epidemics worldwide.

## Competing interests

The authors declare no competing interests.
